# Supplementary material for: All Driven by Energy Demand? Integrative Comparison of Metabolism of Enterococcus faecalis Wildtype and a Glutamine Synthase Mutant
Source: Microbiol Spectr. 2022 Mar 2;10(2):e02400-21. doi: 10.1128/spectrum.02400-21 (PMC8941932; doi:10.1128/spectrum.02400-21)
Supplement: SUPPLEMENTAL FILE 1 — Supplemental material. Download SPECTRUM02400-21_Supp_1_seq7.pdf, PDF file, 0.5 MB [file spectrum02400-21_supp_1_seq7.pdf]

S. Table 1. Time course data showing the flux measurement of glucose, organic acids and amino acids in the course of 21 hours (t1-t8).

Exchange reaction	t1	t2	t3	t4	t5	t6	t7	t8
R_Ex_glc__e	-7.170	-7.036	-7.455	NA	-7.560	NA	-7.881	-9.593
R_Ex_etoh__e	2.157	2.035	2.134	NA	1.899	NA	1.689	1.490
R_Ex_ac__e	4.037	4.385	4.161	NA	2.931	NA	3.314	2.568
R_Ex_lac_L__e	5.878	6.467	7.671	NA	7.339	NA	10.720	15.026
R_Ex_for__e	7.109	7.522	7.262	NA	5.506	NA	5.570	4.226
R_Ex_asp_L__e	-0.127	-0.117	-0.121	-0.105	-0.102	-0.108	-0.092	-0.095
R_Ex_ser_L__e	-0.289	-0.283	-0.296	-0.292	-0.295	-0.317	-0.312	-0.387
R_Ex_glu_L__e	-0.057	-0.045	-0.043	-0.020	-0.014	-0.014	0.010	0.014
R_Ex_gly__e	-0.085	-0.080	-0.087	-0.075	-0.073	-0.077	-0.062	-0.081
R_Ex_his_L__e	-0.016	-0.012	-0.012	-0.006	-0.004	-0.005	0.002	-0.004
R_Ex_nh4__e	0.253	0.274	0.290	0.338	0.363	0.387	0.409	0.530
R_Ex_arg_L__e	-0.284	-0.279	-0.295	-0.294	-0.300	-0.318	-0.312	-0.380
R_Ex_thr_L__e	-0.054	-0.050	-0.054	-0.045	-0.043	-0.045	-0.034	-0.051
R_Ex_ala_L__e	0.060	0.065	0.059	0.080	0.088	0.093	0.117	0.124
R_Ex_pro_L__e	-0.064	-0.057	-0.074	-0.045	-0.039	-0.047	-0.014	-0.043
R_Ex_cys_L__e	0.035	0.034	0.038	0.045	0.045	0.050	0.054	0.068
R_Ex_tyr_L__e	-0.032	-0.022	-0.031	-0.034	-0.013	-0.055	-0.046	-0.045
R_Ex_val_L__e	-0.084	-0.078	-0.085	-0.072	-0.069	-0.074	-0.056	-0.074
R_Ex_met_L__e	-0.021	-0.019	-0.020	-0.017	-0.016	-0.017	-0.013	-0.019
R_Ex_orn_L__e	0.197	0.198	0.207	0.223	0.233	0.246	0.261	0.316
R_Ex_lys_L__e	-0.075	-0.068	-0.076	-0.058	-0.052	-0.057	-0.035	-0.062
R_Ex_ile_L__e	-0.076	-0.073	-0.076	-0.069	-0.067	-0.070	-0.060	-0.073
R_Ex_leu_L__e	-0.134	-0.125	-0.131	-0.114	-0.108	-0.112	-0.087	-0.106
R_Ex_phe_L__e	-0.046	-0.042	-0.046	-0.038	-0.034	-0.040	-0.029	-0.041
R_Ex_asn_L__e	-0.051	-0.049	-0.051	-0.046	-0.046	-0.047	-0.041	-0.052

R_Ex_gln_L__e	-0.147	-0.144	-0.153	-0.152	-0.155	-0.164	-0.162	-0.197
R_Ex_trp_L__e	-0.006	-0.005	-0.006	-0.006	-0.005	-0.006	-0.004	-0.006
R_Ex_citr_L__e	0.022	0.022	0.021	0.022	0.023	0.022	0.023	0.019
R_Ex_cys_L__e	-0.062	-0.060	-0.061	-0.057	-0.055	-0.055	-0.050	-0.053

S. Table 2. Metabolic constraints in the form of lower and upper bounds at pH 7.5.

Exchange reaction	experimental value	calculated lower bound	calculated upper bound	adjusted lower bound	adjusted upper bound
R_Ex_glc__e	-7.170	-8.604	-5.736.000.000.000.000		
R_Ex_etoh__e	2.157	17.256	25.884		
R_Ex_ac__e	4.037	32.296	4.844.399.999.999.990		
R_Ex_lac_L__e	5.878	47.024	70.536		
R_Ex_for__e	7.109	5.687.200.000.000.000	85.308		
R_Ex_asp_L__e	-0.127	-0.1524	-0.10160000000000001		
R_Ex_ser_L__e	-0.289	-0.34679999999999994	-0.2312		
R_Ex_glu_L__e	-0.057	-0.0684	-0.0456		-0.04
R_Ex_gly__e	-0.085	-0.10200000000000001	-0.068		
R_Ex_his_L__e	-0.016	-0.0192	-0.0128	-0.013	0.0
R_Ex_nh4__e	0.253	0.20240000000000002	0.3036		
R_Ex_arg_L__e	-0.284	-0.34079999999999994	-0.22719999999999999		-0.2372
R_Ex_thr_L__e	-0.054	-0.0648	-0.0432		
R_Ex_ala_L__e	0.060	0.048	0.072		0.06
R_Ex_pro_L__e	-0.064	-0.0768	-0.0512	-0.052	0.0
R_Ex_cys_L__e	0.035	0.028000000000000004	0.042		
R_Ex_tyr_L__e	-0.032	-0.0384	-0.0256		
R_Ex_val_L__e	-0.084	-0.1008	-0.06720000000000001		
R_Ex_met_L__e	-0.021	-0.0252	-0.016800000000000002		
R_Ex_orn_L__e	0.197	0.15760000000000002	0.2364		0.2464
R_Ex_lys_L__e	-0.075	-0.09	-0.06		
R_Ex_ile_L__e	-0.076	-0.09119999999999999	-0.0608		
R_Ex_leu_L__e	-0.134	-0.1608	-0.10720000000000002		
R_Ex_phe_L__e	-0.046	-0.0552	-0.0368		

R_Ex_asn_L__e	-0.051	-0.06119999999999999	-0.0408		
R_Ex_gln_L__e	-0.147	-0.17639999999999997	-0.1176		
R_Ex_trp_L__e	-0.006	-0.0072	-0.0048000000000000004		
R_Ex_citr_L__e	0.022	0.0176	0.026399999999999996		0.044
R_Ex_cys_L__e	-0.062	-0.0744	-0.049600000000000005		0.032

S. Table 3. Metabolic constraints in the form of lower and upper bounds at pH 6.5.

Exchange reaction	Experimental value	Calculated lower bound	Calculated upper bound	Adjusted lower bound	Adjusted upper bound
R_Ex_glc__e	-9.593	-115.116	-76.744		
R_Ex_etoh__e	1.490	1.192	1.788		
R_Ex_ac__e	2.568	20.544.000.000.000.000	30.816		
R_Ex_lac_L__e	15.026	12.020.800.000.000.000	180.312		
R_Ex_for__e	4.226	33.808.000.000.000.000	50.712		
R_Ex_asp_L__e	-0.095	-0.11399999999999999	-0.07600000000000001		
R_Ex_ser_L__e	-0.387	-0.4644	-0.30960000000000004		
R_Ex_glu_L__e	0.014	0.011200000000000002	0.0168		
R_Ex_gly__e	-0.081	-0.0972	-0.06480000000000001		
R_Ex_his_L__e	-0.004	-0.0048	-0.0032	-0.0062	
R_Ex_nh4__e	0.530	0.42400000000000004	0.636		
R_Ex_arg_L__e	-0.380	-0.45599999999999996	-0.30400000000000005		
R_Ex_thr_L__e	-0.051	-0.06119999999999999	-0.0408		
R_Ex_ala_L__e	0.124	0.09920000000000001	0.1488		
R_Ex_pro_L__e	-0.043	-0.05159999999999999	-0.0344	-1	3
R_Ex_cys_L__e	0.068	0.054400000000000004	0.0816		
R_Ex_tyr_L__e	-0.045	-0.054	-0.036		
R_Ex_val_L__e	-0.074	-0.08879999999999999	-0.0592		
R_Ex_met_L__e	-0.019	-0.022799999999999997	-0.0152		
R_Ex_orn_L__e	0.316	0.2528	0.3792		
R_Ex_lys_L__e	-0.062	-0.0744	-0.049600000000000005		
R_Ex_ile_L__e	-0.073	-0.0876	-0.0584		
R_Ex_leu_L__e	-0.106	-0.12719999999999998	-0.0848		

R_Ex_phe_L__e	-0.041	-0.0492	-0.0328		
R_Ex_asn_L__e	-0.052	-0.0624	-0.0416		
R_Ex_gln_L__e	-0.197	-0.2364	-0.15760000000000002		
R_Ex_trp_L__e	-0.006	-0.0072	-0.0048000000000000004		
R_Ex_citr_L__e	0.019	0.0152	0.022799999999999997		
R_Ex_cys_L__e	-0.053	-0.06359999999999999	-0.0424		0

S. Table 4. List of inactivated genes following the integration of proteome data at pH 7.5.

**Reactions**	**Genes**	**Reactions**	**Genes**
R_TAL	EF3304	R_FRDx	EF2566
R_LDH_D	EF2295	R_ARABte	EF2773
R_MALt	EF1920	R_PNTOt2	EF2657
R_SERt6	EF3015	R_THMASE	EF2767
R_DALAt	EF1103	R_RMK	EF0433
R_TMDPK	EF3117	R_RMI	EF0434
R_RIBFLVt2	EF1541	R_GARFT	EF1779
R_HXANt2	EF2935	R_VANX	EF2293
R_MENE	EF0446		
R_ARGORNt	EF0108		
R_LYSt6	EF0929		
R_XYLB	EF0557		
R_XYLA	EF0556		
R_XYLI	EF0557		
R_FUMt	EF1920		
R_PTB	EF1663		
R_RIBt2	EF2959		
R_GLNS	EF2159		
R_NH4t	EF0547		
R_UNAGAMAMT	EF1173		
R_RAFDH	EF1603		
R_CYSTGL	EF3284		
R_DSERt	EF1103		
R_PIht6	EF2442		

S. Table 5. List of reactivated genes for the model to have a feasible solution (pH 7.5).

**Genes**
EF0387
EF3069
EF0108
EF0929
EF0547
EF2442
EF3015
EF3199
EF3200
EF3117

S. Table 6. List of proteomic constraints in the form of reactions upper and lower bounds at pH 6.5.

Reactions	Adjusted lower bound	Adjusted upper bound
R_PRFGS	0	0
R_SPMDabc	0	0
R_PDE1	0.0	0.0
R_PDE2	0.0	0.0
R_PDE3	0.0	0.0
R_PDE4	0.0	0.0
R_PDE5	0.0	0.0
R_NDPK9	0.0	0.0
R_NDPK8	0.0	501130
R_NDPK7	0.0	0.0010203525938033196
R_NDPK6	0.0	0.0013224859790384868
R_NDPK5	0.0	0.0010203525938033196
R_GLNabc	0.0	0.06837444461554447
R_GMPR	0.0	0.000768957299464954
R_XANt2	0.002012843715119238	0.005257190576108279
R_IMPC	0.0	0.0
R_ME1x	0.0	0.0
R_AIRC	0.0	0.0
R_FMETDF	0.0	0.0
R_TREpts	0.0	0.0
R_ADSS	0.0	0.0
R_GLUPRT	0.0	0.0
R_URAt2	0.0003167241962024313	0.011771168317517122
R_AICART	0.0	0.0
R_XPPT	0.002720007446909017	0.007104176766568783

R_PMANM	0.0	0.0
R_PTRCabc	0.0	0.0
R_CDD	0.0	0.0
R_GLYK	0.0	0.0024090417384447812
R_PRAGS	0.0	0.0
R_MALPP	0.0	0.0
R_OAADC	0.0	0.011374005166922309
R_PRAIS	0.0	0.0

S. Table 7. List of missing essential genes from the experimentally detected protein library.

Genes	Reactions
EF0168	R_PNTK
EF0739	R_NACUP, R_NCAMRNSt
EF0904	R_MEVK
EF0992	R_PAPPT1_A, R_PAPPT1_L
EF1391	R_PZS
EF1392	R_PZS
EF1393	R_PZS
EF1396	R_PZS, R_Moabc
EF2183	R_LTAS2
EF2294	R_VANB
EF2411	R_DAGK_LPL
EF2439	R_UDCPK, R_UDCPDP
EF2494	R_DASYN_LPL
EF2661	R_DAGK_LPL
EF2746	R_DARTAL_EFA, R_DALTAL_EFA
EF2748	R_DARTAL_EFA, R_DALTAL_EFA
EF2973	R_ALKP_Efa
EF3072	R_BTNt2i

S. Table 8. List of essential reactions (reactions that are necessary for the model to have a feasible solution.)

R_ACACT1	R_UAGPT1_A	R_DPMVD	R_HBUHL1	R_TMDS	R_LEUt6
R_ACCOAC	R_UAGPT1_L	R_DTMPK	R_HBUR1	R_TRDR	R_LEUTRS
R_AGAT_EFA	R_UAMAGS	R_EPA_PS_EFA	R_HCO3E	R_TRPTRS	R_LPGS_EFA
R_ALAR	R_UAMAS	R_Ex_arg_L__e	R_HDDHL5	R_TYRTRS	R_LTAS1
R_ALATA_Lr	R_UAPGR	R_Ex_btn__e	R_HDDR5	R_HISTRS	R_LTAS2
R_ALATRS	R_UDCPDP	R_Ex_glyclt__e	R_HDEHL4	R_HMGCOAR	R_LYSTRS
R_ALKP_Efa	R_UDCPDPS	R_Ex_his_L__e	R_HDER4	R_HMGCOAS	R_MACPMT
R_ARGTRS	R_UDPG4E	R_Ex_ile_L__e	R_HDMAT7	R_HOCHL3	R_MCMAT2
R_ASPTRS	R_UGLDDS1_A	R_Ex_leu_L__e	R_HEMAT2	R_HOCR3	R_MCMAT3
R_BIOMASS	R_UGLDDS1_L	R_Ex_nac__e	R_HHDHL7	R_HODHL8	R_MCMAT4
R_BPPA1	R_URIDK1	R_Ex_pydam__e	R_HHDR7	R_HODR8	R_MCMAT5
R_BPPA1_L	R_VALt6	R_Ex_thm__e	R_HHYHL2	R_HPPK	
R_BPPA2	R_VALTRS	R_Ex_trp_L__e	R_HHYR2	R_HTDHL6	
R_BPPA2_L	R_VANB	R_Ex_val_L__e	R_HISt6	R_HTDR6	
R_BTMAT1	R_WTASI	R_FABM1	R_MCMAT6	R_ILEt6	
R_BTNt2i	R_WTASII	R_FABM2	R_MCMAT7	R_ILETRS	
R_CLPNS_LPL	R_DARTAL_EFA	R_G1PACT	R_MCMAT8	R_IPDDI	
R_CPS_EFA_SYNTH	R_DASYN_LPL	R_G1PTMT	R_PYDAMt	R_kaasIII	
R_CPS_PS_EFA	R_DDL	R_GALU	R_PYDXK	R_LEUt6	
R_CRCT	R_DDMAT5	R_GAT1_EFA	R_PZS	R_LEUTRS	
R_CYSTRS	R_DEMAT4	R_GCALDD	R_RBT5PDHy	R_LPGS_EFA	
R_CYTK1	R_DEX_PS_EFA	R_GF6PTA	R_RNAS_LPL	R_LTAS1	
R_DAGGT_LPL	R_DHFR	R_GLNTAL	R_SERTRS	R_LTAS2	
R_DAGK_LPL	R_DHFS	R_GLUR	R_TAPGL4_EFA	R_LYSTRS	
R_DALTAL_EFA	R_DHNPA	R_GLUTRS	R_TDMAT6	R_MACPMT	
R_UAAGLS1	R_DHPS3	R_GLYCLTt2r	R_TDPDRE	R_MCMAT2	
R_UACGE	R_DMATT	R_GLYTRS	R_TDPGDH	R_MCMAT3	
R_UAGCVT	R_DNAS_LPL	R_GRTT	R_THMabc	R_MCMAT4	
R_UAGDP	R_DPCOAK	R_GTPCI	R_THRTRS	R_MCMAT5	

S. Table 9. List of essential genes and their respective enzymes.

Genes	Enzyme
EF0043	Glutamyl-tRNA synthetase
EF0045	Cysteinyl-tRNA synthetase (Cysteine--tRNA ligase) (CysRS)
EF0059	glucosamine-1-phosphate-N-acetyltransferase
EF0090	Diacylglycerol kinase (LPL specific)
EF0168	pantothenate kinase
EF0268	Lysyl-tRNA synthetase
EF0680	peptidoglycan glycosyltransferase
EF0724	glutamyl-tRNA(Gln):L-glutamine amido-ligase (ADP-forming)
EF0725	glutamyl-tRNA(Gln):L-glutamine amido-ligase (ADP-forming)
EF0726	glutamyl-tRNA(Gln):L-glutamine amido-ligase (ADP-forming)
EF0727	Diacylglycerol kinase (LPL specific)
EF0739	Nicotinic acid uptake
EF0770	Phosphatidylglycerol phosphate phosphatase (LPL specific)
EF0801	Leucyl-tRNA synthetase
EF0843	D-alanine—D-alanine ligase (reversible) (D-Ala-D-Ala ligase) (D-alanylalanine synthetase)
EF0845	UDP-N-acetylmuramoyl-L-alanyl-D-glutamyl-L-lysyl-D-alanyl-D-alanine synthetase (alpha-glutamate)
EF0849	alanine racemase
EF0880	dephospho-CoA kinase
EF0901	isopentenyl-diphosphate D-isomerase
EF0902	phosphomevalonate kinase
EF0903	diphosphomevalonate decarboxylase
EF0904	mevalonate kinase
EF0930	Methionyl-tRNA synthetase
EF0981	geranyltranstransferase
EF0991	peptidoglycan glycosyltransferase

EF0992	phospho-N-acetylmuramoyl-pentapeptide-transferase (alpha-glutamate) (D-ala)
EF0993	UDP-N-acetylmuramoyl-L-alanyl-D-glutamate synthetase
EF0994	UDP-N-acetylglucosamine-N-acetylmuramyl-(pentapeptide)pyrophosphoryl-undecaprenol N-acetylglucosamine transferase
EF1003	Isoleucyl-tRNA synthetase
EF1036	nucleoside-diphosphate kinase (ATP:GDP)
EF1115	Phenylalanyl-tRNA synthetase (Phenylalanyl-tRNA synthetase alpha subunit)
EF1116	Phenylalanyl-tRNA synthetase (Phenylalanyl-tRNA synthetase beta subunit)
EF1121	glutamate racemase
EF1148	peptidoglycan glycosyltransferase
EF1364	Hydroxymethylglutaryl CoA reductase
EF1379	Alanyl-tRNA synthetase (AlaRS)
EF1391	precursor Z synthase
EF1392	precursor Z synthase
EF1393	precursor Z synthase
EF1396	precursor Z synthase
EF1547	cytidylate kinase (dCMP)
EF1576	thymidylate synthase
EF1577	dihydrofolate reductase
EF1711	carbonate dehydratase (HCO3 equilibration reaction)
EF1746	UTP-glucose-1-phosphate uridylyltransferase
EF1908	UDP-N-acetylmuramoyl-L-alanine synthetase
EF1970	Aspartyl-tRNA synthetase (AspRS)
EF1971	Histidyl-tRNA synthetase
EF2150	UDP-N-acetylglucosamine 4-epimerase
EF2162	dimethylallyltranstransferase
EF2182	Lipoteichoic acid synthase (LPL specific)
EF2183	Lipoteichoic acid modification (koijbiose)

EF2192	dTDPglucose 4
EF2193	dTDP-4-dehydromannose 3
EF2194	glucose-1-phosphate thymidylyltransferase
EF2294	D-alanine—(R)-lactate ligase
EF2379	Prolyl-tRNA synthetase
EF2396	uridylate kinase (UMP)
EF2406	Glycyl-tRNA synthetase
EF2407	Glycyl-tRNA synthetase
EF2411	Diacylglycerol kinase (LPL specific)
EF2439	Undecaprenyl-diphosphatase
EF2451	pantetheine-phosphate adenylyltransferase
EF2471	Arginyl-tRNA synthetase (ArgRS)
EF2476	peptidoglycan glycosyltransferase
EF2494	CDP-Diacylglycerol synthetase (Phosphatidate cytidylyltransferase) (LPL specific)
EF2495	Undecaprenyl diphosphate synthase
EF2644	Diacylglycerol kinase (LPL specific)
EF2655	phosphopantothenate-cysteine ligase
EF2656	phosphopantothenoylcysteine decarboxylase
EF2658	UDP-N-acetylmuramoylpentapeptide-lysine-N6-alanyltransferase
EF2661	Diacylglycerol kinase (LPL specific)
EF2691	1-Acyl-glycerol-3-phosphate acyltransferase (Efa specific)
EF2746	D-Alanine lipoteichoic acid ligase
EF2747	D-Alanine lipoteichoic acid ligase
EF2748	D-Alanine lipoteichoic acid ligase
EF2749	D-Alanine lipoteichoic acid ligase
EF2764	Thymidylate kinase (dTMP kinase)
EF2858	Threonyl-tRNA synthetase

EF2871	nicotinate-nucleotide adenylyltransferase (nicotinate)
EF2875	acetyl-CoA carboxylase
EF2876	acetyl-CoA carboxylase
EF2877	acetyl-CoA carboxylase
EF2879	acetyl-CoA carboxylase
EF2882	Malonyl-CoA:[acyl-carrier-protein] S-malonyltransferase
EF2883	Tetradecanoyl-[acyl-carrier protein]:malonyl-CoA C-acyltransferase
EF2885	beta-ketoacyl-ACP synthase III
EF2890	1
EF2891	1
EF2908	peptidoglycan glycosyltransferase
EF2928	dihydrofolate synthase
EF2931	Valyl-tRNA synthetase
EF2973	alkaline phosphatase
EF3072	Biotin uptake
EF3112	glycerol 3-phosphate acyltransferase (Efa specific)
EF3129	peptidoglycan glycosyltransferase
EF3148	lysylphosphatidyl-glycerol synthetase
EF3265	dihydropteroate synthase
EF3267	GTP cyclohydrolase I
EF3268	2-amino-4-hydroxy-6-hydroxymethyldihydropteridine diphosphokinase
EF3269	dihydroneopterin aldolase

S. Table 10. Significant fold changes at the proteome level between t3 and t1.

Reaction abbreviation	Change	p-value
R_CTPS2	1.63890193589	0.04828668
R_PMEVK	1.97630931519	0.049963774
R_TAGA	1.71091285581	0.048401574
R_FMNAT	1.53450840082	0.018418951
R_NNAMr	0.579906671149	0.043743765
R_RNDR2	0.113556516552	0.014524055
R_ASPCT	0.540883578382	0.014436519
R_DEX_PS_EFA	0.677245103099	0.034169764
R_G3PO	2.93224651611	0.049505943
R_OCBT	0.540883578382	0.014436519
R_GMPR	0.580492532748	0.044735034
R_SBTpts	0.208542436348	0.010564345
R_DHNAS	1.54800580009	0.042118513
R_PGL	0.620953374912	0.016626571
R_MALTabc	2.71542379428	0.014436519
R_ARGD	1.57686937955	0.038619556
R_TYRTA	1.83546731126	0.030302482
R_CDPMEK	2.9629291135	0.042488016
R_RNDR4	0.113556516552	0.014524055
R_RNDR3	0.113556516552	0.014524055
R_PGPP_LPL	0.486269996416	0.014436519
R_TAPGL4_EFA	0.540883578382	0.014436519
R_DAPDC	1.60416754956	0.031295061
R_DHFOR2	0.444448732256	0.042674702
R_TDPDRE	0.526923889232	0.040338473
R_UACGE	0.677245103099	0.034169764
R_DHFR	0.444448732256	0.042674702
R_VALTRS	2.33769302453	0.032222324
R_GTHS	2.02787326816	0.031295061
R_CDD	0.486664770062	0.00041297
R_RNDR1	0.113556516552	0.014524055

R_CELBpts	0.532183444906	0.004964827
R_CELBpts	0.532183444906	0.004964827
R_BPPA2_L	0.677245103099	0.034169764
R_RPI	2.73601994462	0.043937668
R_GLUCYSL	2.02787326816	0.031295061
R_EPA_PS_EFA	0.677245103099	0.034169764
R_ALATA_L	2.46626089459	0.04926589
R_ACTPASE	0.543284194457	0.014436519
R_BPPA2	0.677245103099	0.034169764
R_PANB	1.54123791035	0.031265045
R_G1PACT	2.26209396876	0.029276069
R_UAGDP	2.26209396876	0.029276069
R_RBFK	1.53450840082	0.018418951
R_MTHPTGHM	2.01200124101	0.045724593
R_GHMT	2.31513631222	0.031295061

S. Table 11. Significant fold changes at the proteome level between t4 and t1.

Reaction abbreviation	Change	p-value
R_PGL	0.5738456585	0.019662016
R_CDD	0.58433750945	0.008558951
R_ACTPASE	0.543462288312	0.031954297

S. Table 12. Significant fold changes at the proteome level between t5 and t1.

Reaction abbreviation	Change	P-value
R_CDD	0.584205462912	0.011112227
R_ACTPASE	0.523644783934	0.025410243

S. Table 13. Significant fold changes at the proteome level between t6 and t1.

Reaction abbreviation	Change	P-value
R_CDD	0.521029799606	0.001808714
R_ACTPASE	0.477016117438	0.009193028

S. Table 14. Significant fold changes at the proteome level between t7 and t1.

Reaction abbreviation	Change	P-value
R_CDD	0.526602135869	0.004403881
R_ACTPASE	0.544365914365	0.032780414

S. Table 15. Significant fold changes at the proteome level between t8 and t1.

Reaction abbreviation	Changes	p-value
R_PRPPS	0.702185299835	0.01759182
R_PRFGS	0.294343721566	0.000171526
R_SPMDabc	0.128708671432	0.000891361
R_PDE1	0.579410399883	0.011248306
R_PDE2	0.579410399883	0.011248306
R_PDE3	0.579410399883	0.011248306
R_PDE4	0.579410399883	0.011248306
R_PDE5	0.579410399883	0.011248306
R_NDPK9	0.357950631741	0.042018929
R_NDPK8	0.357950631741	0.042018929
R_NDPK7	0.357950631741	0.042018929
R_NDPK6	0.357950631741	0.042018929
R_NDPK5	0.357950631741	0.042018929
R_NDPK4	0.357950631741	0.042018929
R_NDPK3	0.357950631741	0.042018929
R_NDPK2	0.357950631741	0.042018929
R_NDPK1	0.357950631741	0.042018929
R_GLNabc	1.55907771659	0.003943372
R_GMPR	0.457885582652	0.006045753
R_XANt2	0.379306679373	0.011248306
R_IMPC	0.116277843614	1.52e-05
R_ME1x	0.615708970929	0.018231213
R_AIRC	0.228685137252	0.000929637
R_FMETDF	1.6307598154	0.023588934
R_PGAMT	0.355650632158	0.009695126
R_TREpts	0.280736842019	5.44e-05
R_ADSS	0.406572914915	2.98e-05
R_GLUPRT	0.337390147352	0.023338551
R_URAt2	0.379306679373	0.011248306
R_AICART	0.116277843614	1.52e-05

R_XPPT	0.512566866275	0.0159729
R_PMANM	0.355650632158	0.009695126
R_PTRCabc	0.128708671432	0.000891361
R_PGMT	0.355650632158	0.009695126
R_CDD	0.395281255682	8.09e-06
R_GLYK	0.591527293003	0.026602872
R_PRAGS	0.366141109905	0.024864028
R_MALPP	0.564735962708	0.023338551
R_OAADC	0.615708970929	0.018231213
R_PRAIS	0.331714599531	0.010924227

S. Table 16. Flux measurement of glucose, organic acids and amino acids at two dilatation rates, 0.15 and 0.05 h^{-1} at pH 7.5 and 6.5. The data is used for the estimation of ATP maintenance.

**Reactions**	**Experimental value**			
	7,5, 0,15	6,5, 0,15	7,5 0,05	6,5, 0,05
R_Ex_glc__e	-6,3230357	-8,74125	-4,1436652	-5,2328571
R_Ex_etoh__e	3,00853211	1,31258619	1,954819	1,43534594
R_Ex_ac__e	3,24047722	1,6646415	2,34504525	1,81889951
R_Ex_lac_L__e	7,54292064	15,9580439	2,60823222	5,56738437
R_Ex_for__e	5,57835409	2,79945752	4,41502043	3,30368615
Succinate	0	0	0	0
R_Ex_pyr__e	0,10182884	0,10656909	0,09474662	0,12763664
Acetoin	0	0	0	0
2.3-butanediol	0	0	0	0
CO2	0	0	0	0
O2	0	0	0	0
R_Ex_asp_L__e	-0,1308159	-0,137878	-0,1313828	-0,1505571
R_Ex_ser_L__e	-0,3023771	-0,3221543	-0,1854702	-0,2508137
R_Ex_glu_L__e	-0,0824365	-0,0705838	-0,0275192	-0,0733358
R_Ex_gly__e	-0,0953275	-0,0597635	-0,0581162	-0,0977894
R_Ex_his_L__e	-0,0333229	-0,0289961	-0,0186223	-0,0300862
R_Ex_nh4__e	-0,5438415	-0,751831	-0,3563948	-0,4500757
R_Ex_arg_L__e	-0,2994718	-0,4119674	-0,121859	-0,2481603
R_Ex_thr_L__e	-0,0737675	-0,0530418	-0,0425567	-0,0646863
R_Ex_ala_L__e	0,00692002	0,15424036	-0,000855	0,00952816
R_Ex_pro_L__e	0,0554295	0,16986676	-0,0973449	-0,1101082
R_Ex_cys_L__e	-0,0859643	-0,1188409	-0,0563348	-0,0711429
R_Ex_tyr_L__e	-0,0770671	-0,1081494	-0,0298488	-0,0621639

R_Ex_val_L__e	-0,1156056	-0,1069205	-0,0658251	-0,1054554
R_Ex_met_L__e	-0,0339244	-0,038035	-0,0570136	-0,0515313
Ornithine	0	0	0	0
R_Ex_lys_L__e	-0,043371	-0,0116138	-0,0139551	-0,0388254
R_Ex_ile_L__e	-0,0906547	-0,0875255	-0,0409405	-0,0770522
R_Ex_leu_L__e	-0,1538447	-0,1398334	-0,0807965	-0,1387774
R_Ex_phe_L__e	-0,0653184	-0,078708	-0,0400491	-0,0649809
R_Ex_trp_L__e	-0,0071603	-0,0063578	-0,0165611	-0,007572
R_Ex_asn_L__e	-0,0527041	-0,0388719	-0,0513122	-0,0335545
R_Ex_gln_L__e	-0,1416857	-0,1958727	-0,0928507	-0,1172571

ATPM-estimation

S. Table 17. Estimation of ATPM value using the linear function fitted to the estimated values at different dilution rates.

		Growth rate		
		0,15	0,05	0
		**Optimized value**		**Estimated value**
**pH**	7,5	10,761	7,572	5,977
	6,5	13,680	8,709	6,224

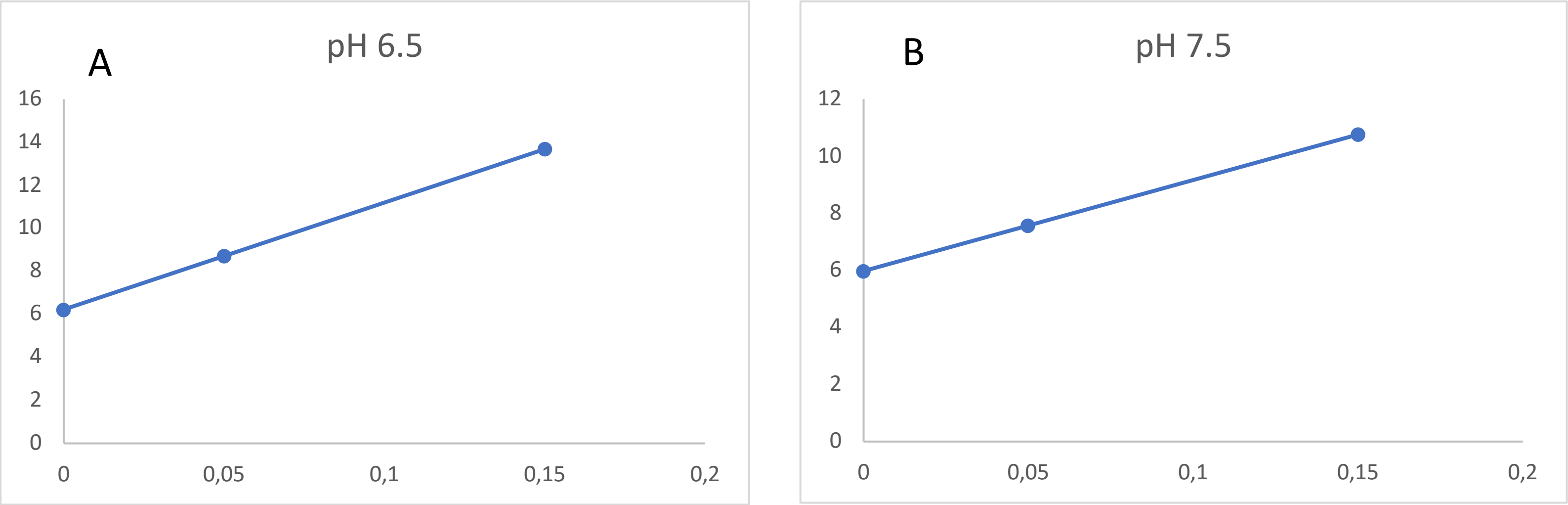

S- Figure 1.The linear line fitting the non-growth associated ATP at different dilution rates. The y-intercept is the ATPm value, representing the required maintanace energy at zero growth rate.
